# Overlaps and divergences between tauopathies and synucleinopathies: a duet of neurodegeneration

**DOI:** 10.1186/s40035-024-00407-y

**Published:** 2024-03-26

**Authors:** Wen Li, Jia-Yi Li

**Affiliations:** 1 Health Sciences Institute, Key Laboratory of Major Chronic Diseases of Nervous System of Liaoning Province, China Medical University, Shenyang, 110122 China; 2 Neural Plasticity and Repair Unit, Department of Experimental Medical Science, Wallenberg Neuroscience Center, Lund University, BMC A10, 22184 Lund, Sweden

**Keywords:** Tauopathy, Synucleinopathy, Tau, α-Synuclein, Neurodegeneration

## Abstract

Proteinopathy, defined as the abnormal accumulation of proteins that eventually leads to cell death, is one of the most significant pathological features of neurodegenerative diseases. Tauopathies, represented by Alzheimer’s disease (AD), and synucleinopathies, represented by Parkinson’s disease (PD), show similarities in multiple aspects. AD manifests extrapyramidal symptoms while dementia is also a major sign of advanced PD. We and other researchers have sequentially shown the cross-seeding phenomenon of α-synuclein (α-syn) and tau, reinforcing pathologies between synucleinopathies and tauopathies. The highly overlapping clinical and pathological features imply shared pathogenic mechanisms between the two groups of disease. The diagnostic and therapeutic strategies seemingly appropriate for one distinct neurodegenerative disease may also apply to a broader spectrum. Therefore, a clear understanding of the overlaps and divergences between tauopathy and synucleinopathy is critical for unraveling the nature of the complicated associations among neurodegenerative diseases. In this review, we discuss the shared and diverse characteristics of tauopathies and synucleinopathies from aspects of genetic causes, clinical manifestations, pathological progression and potential common therapeutic approaches targeting the pathology, in the aim to provide a timely update for setting the scheme of disease classification and provide novel insights into the therapeutic development for neurodegenerative diseases.

## Background

Tauopathies are defined as a spectrum of neurodegenerative diseases with pathological features of intracellular (neuronal or glial) deposition of hyperphosphorylated tau, forming neurofibrillary tangles (NFTs) [1]. So far, more than 26 neurological diseases have been identified as tauopathies, including Alzheimer’s disease (AD), Pick’s disease, and progressive supranuclear palsy (PSP), manifesting symptoms of dementia and motor deficits [2]. Tauopathies can be classified as primary or secondary depending on whether other proteinopathies are involved, such as extracellular Aβ plaque formation. For example, frontotemporal dementia (FTD), in which tau is the dominant composition of protein aggregates, is a primary tauopathy [3, 4]. On the contrary, AD is a secondary tauopathy, possessing both NFTs and Aβ plaques [2, 5].

Certain series of neurodegenerative diseases can be pathologically characterized by their common “driving-force” proteins, such as tauopathies by tau [6]. Similarly, synucleinopathies including Parkinson’s disease (PD), Lewy body dementia (DLB) [7] and multiple system atrophy (MSA) are characterized by aggregation of α-synuclein (α-syn) [8]. Based on the cellular deposition of α-syn, synucleinopathies can be categorized into Lewy body diseases with α-syn inclusions in neurons forming Lewy bodies (LB) and Lewy neurites (LN), and MSA with α-syn aggregation in glia forming glial cytoplasmic inclusions [9]. Synucleinopathies mainly manifest motor deficits appearing as parkinsonism, such as tremor, rigidity, and bradykinesia in PD. However, in the early stages of diseases, non-motor symptoms such as rapid eye movement sleep behavior disorder are also present. Meanwhile, cognitive decline is also observed in synucleinopathies such as DLB [10].

Genome-wide association studies have revealed that synucleinopathies and tauopathies share genetic risks such as *MAPT* (encoding microtubule-associated protein tau) mutations [11–15]. Also, association between *SNCA* (α-syn-encoding gene) polymorphism and increased risk of AD has been reported [16]. A known risk locus of AD, apolipoprotein E (*APOE)*, is also a genetic risk factor for DLB [17, 18]. The *APOE* allele status has been associated with both PD onset and its progression to Parkinson’s disease dementia (PDD) [19, 20]. Clinically, familial FTD (FTDP-17) exhibits motor symptoms of parkinsonism, while 83% of PD cases progress into PDD in the late stage [8, 21–23]. Pathologically, tau and α-syn have been shown to cross-seed each other in the progression of aggregation formation [24]. In summary, tauopathies and synucleinopathies have overlapping characteristics in etiology, pathology and clinical manifestations. The interacting and independent features of both pathological proteins and groups of diseases may indicate the possibility of developing differential diagnosis and pathology-targeting therapeutic interventions.

In this review, we summarize the overlapping and the divergent features of tauopathies and synucleinopathies, from perspectives of genetic risk to pathological development, with a special focus on pathology continuum between tau and α-syn aggregation. Furthermore, we discuss possible immunotherapies targeting pathology spreading that can be used for both tauopathies and synucleinopathies. A summary of examples of tauopathies and synucleinopathies and their pathological features is presented in Table 1.

**Table 1:** Examples of tauopathies and synucleinopathies- clinical manifestation and pathological features

Type of proteinopathy	Disease	Clinical manifestation	Protein/cell type/ pathology	Main affected regions in the CNS and PNS
Synucleinopathy	Parkinson's disease [25]	Non-motor symptoms, tremor, rigidity, bradykinesia	α-syn/neurons/Lewy bodies (LBs) and Lewy neurites (LNs)	Substantia nigra, basal ganglia
	Lewy body dementia [26, 27]	Visual hallucinations, cognitive decline, difficulty walking, rigidity	α-syn/neurons/LBs and LNs	Cortical and subcortical region
	Multiple system atrophy [28, 29]	Slowness of movement, stiffness, cerebellar ataxia, autonomic failure	α-syn/oligodendrocytes/GCIs	Basal ganglia, cerebellum, pons, spinal cord
	Pure autonomic failure [30]	Orthostatic hypotension, autonomic failure, RBD	α-syn/neurons/LBs and LNs	Autonomic nerves and ganglia, nigra, locus coeruleus
Primary tauopathy	Cortiobasal degeneration [31]	Apraxia, dystonia, lack of balance, stiffness, dementia	4R tau/glia and neurons/ballooned neurons, pretangles, coiled bodies	Cortex and basal ganglia
	Pick's disease [32]	Dementia with frontotemporal degeneration	3R tau/glia and neurons/Pick bodies, ballooned neurons	Cortex and hippocampus
	Progressive supranuclear palsy [33]	Ocular motor dysfunction, postural instability, akinesia, dementia	4R tau/glia and neurons/NFTs, globose tangles, tufted astrocytes, coiled bodies	Cortex, basal ganglia, brainstem
	Chronic traumatic encephalopathy [34, 35]	“Dementia pugilistica” Headaches, dementia, abnormal gait, depression, related to TBI	3R/4R tau/neurons and glia/NFTs	Cortex, basal ganglia, brainstem, depths of cerebral sulci
	Primary age-related tauopathy [36]	“Tangle-only dementia”	3R/4R tau/neurons/NFTs	Cortex, brainstem, olfactory bulb
Secondary tauopathy	Alzheimer's disease [37]	Dementia, with random motor deficits and personality changes	3R/4R tau/neurons/NFTs, plaques, neuropil threads	Hippocampus, limbic, entorhinal cortex, neocortex

## Genetic overlap between tauopathies and synucleinopathies

### *MAPT* in tauopathies

**Fig. 1:**
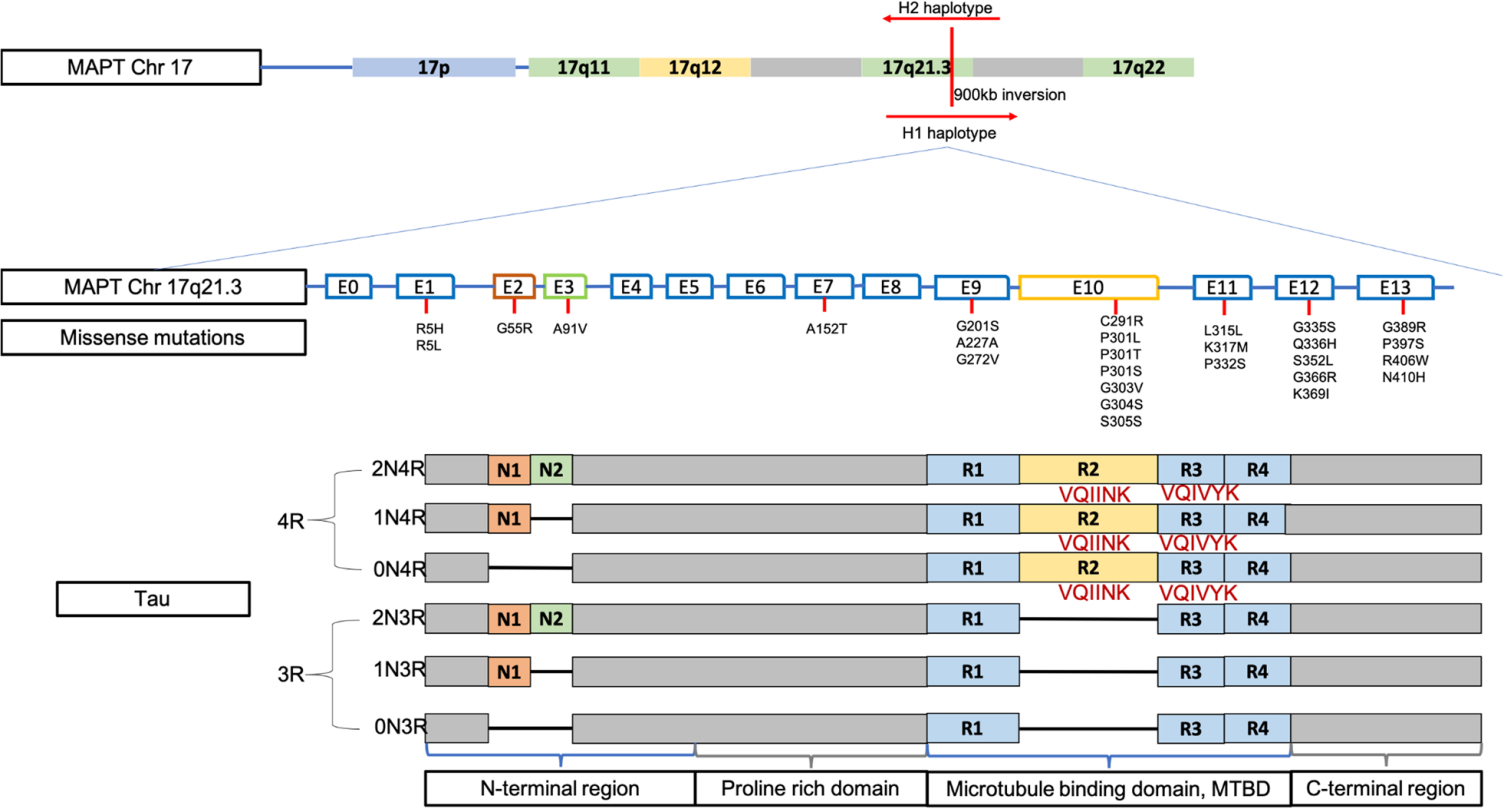
A schematic showing mutations of *MAPT* and composition of tau isoforms. The H1 and H2 haplotypes are formed by the 900 kb inversion in the q21.3 region of chromosome 17. The H1 haplotype is often the one contributing to disease initiation due to multiple missense mutations from exon 1 to 13, especially on exon 10. The tau protein can be classified into 6 isoforms depending on the number of amino inserts (0N, 1N, 2N) on exons 2 and 3 and the number of microtubule-binding domains (3R, 4R). Exon 10 encodes the R2 domain, and its alternative splicing produces 3R or 4R tau isoforms. The microtubule-binding domain contains hexapeptide motifs VQIINK in R2 and VQIVYK in R3. Interactions between the two motifs promote dimer formation of tau [1, 2, 73]

The *MAPT* gene is located on chromosome 17q21.31 and consists of 15 exons. *MAPT* has two haplotypes due to the inversion of the sequence on the 17q21 chromosome [38]. The H1 haplotype is more frequent in humans [39]. The alternative splicing of *MAPT* exons gives rise to six isoforms of tau protein depending on the number of microtubule-binding repeat domains (MTBD) (3R, 4R) and N-terminal inserts (0N, 1N, 2N) [40, 41] (Fig. 1). Over 50 mutations in *MAPT* have been identified to be related to neurodegenerative diseases, most of which are missense mutations [42, 43]. Around 30% of primary tauopathy cases are associated with pathogenic mutations of *MAPT* [43]. In the coding regions of *MAPT*, the P301L mutation on exon 10 is the most prevalent. It has been shown to cause an inherited form of FTD, and induce pathogenesis in FTD, corticobasal syndrome (CBS) and globular glial tauopathies [44, 45]. In addition to the autosomal dominant mutations, *MAPT* variants also serve as a risk factor for primary tauopathies. In sporadic PSP, *MAPT* mutations such as P301L, R5L and S285R are the strongest genetic risk factors [46–48]. In CBS, *MAPT* is the second most common genetic risk factor, only next to the progranulin-coding gene [49]. *MAPT* mutations mainly induce downstream *MAPT* mRNA splicing deficiency and structural changes in tau protein, leading to weak binding of tau to microtubule, alterations of the 4R/3R ratio and the sequential tau aggregation [50].

### *SNCA* in synucleinopathy

The *SNCA* gene is mapped to chromosome 4q22.1 and consists of 6 exons with the last 5 translatable to α-syn protein, which is pathologically responsible for synucleinopathies [51]. The *SNCA* gene plays a prominent role in the onset of synucleinopathies especially PD, both as a causative gene and as risk variants [52]. Similar to *MAPT*, exon splicing of *SNCA* gives rise to alternative transcripts. Both missense mutation and gene multiplication of *SNCA* play important roles in pathology initiation. In the 1990s, the A53T autosomal dominant mutation was found to be related to the early onset of PD in the Contursi Kindred [53]. *SNCA* genetic variants such as E46K [54], H50Q [55], G51D [56] and A30P [57], have been revealed to be associated with the familial forms of synucleinopathies, especially PD. Point mutations such as G51D and multiplication of *SNCA* can promote formation of the amyloid structure of α-syn and facilitate the progression of pathology in synucleinopathies [54, 56, 58, 59]. Thus, gene coding dosage mutations play an important role in the progress of synucleinopathies. *SNCA* triplication leads to early-onset PD with symptoms of dementia [59], while *SNCA* duplication is associated with late-onset PD [58].

### *MAPT* and *SNCA* correlate in the onset of proteinopathies

*MAPT* and *SNCA* gene variants have been shown to be correlated with each other in the etiology of various proteinopathies. First, synucleinopathy and tauopathy have been detected with overlapping presence of mutations of the two genes. Meta-analyses of genome-wide association studies have repeatedly spotted *MAPT* H1 haplotype as a risk locus for PD onset [60]. Although not among the entire spectrum of diseases, certain tauopathies and synucleinopathies have been shown to share genetic risks. AD and DLB, while both presenting dementia as the main clinical symptom, share *APOE* and *BIN1* (bridging integrator-1) as risk genetic loci [18]. Genome-wide analysis revealed that the global genetic correlation between AD and DLB is as high as 0.578, while that between PD and AD is 0.08 [61]. The correlation between genetic risks of AD and ALS is also significant, with shared locus of *TSPOAP1-AS1* [62]. The *MAPT* inversion polymorphism increases the risk of PD and is strongly associated with the development of dementia among PD patients [63]. Carriers of *LRRK2* (leucine-rich repeat kinase 2) mutations, which are the most common cause of familial PD, show abundant tau deposition [64]. There are also cases of *MAPT* mutation working independently of *SNCA* to cause Parkinsonism, showing the possibility that some tauopathies may share pathogenic pathways with synucleinopathies [65, 66].

Besides the direct overlaps between *MAPT* and *SNCA*, tauopathies and synucleinopathies also share common pathogenic pathways [67]. *SNCA* gene point mutations are associated with impairment of key cellular functions, such as tubulin binding, as some point mutations of *SNCA* map to the putative tubulin-binding site, paving the way to tauopathies [68]. In addition, two genes, *MAPT* and *HLA* (human leukocyte antigen), and 10 pathways such as proteolytic signatures, overlap between AD and PD [69, 70]. Studies have shown that the tauopathy-related *MAPT* variants and the synucleinopathy-associated *SNCA* mutation may make neurons more vulnerable to cellular dysfunction such as mitochondrial deficits, setting similar mechanistic paths towards neurodegenerative cell death [71].

## Structural basis for the formation of tau and α-syn pathology

### Structural basis for tau aggregation

The six tau protein isoforms produced by alternative mRNA splicing of *MAPT* range from 352 to 441 amino acids [72]. Tau has physiological functions of stabilizing microtubules and maintaining axonal transport in highly polarized neurons [73]. The N-terminus of tau directly binds to the microtubules, laying the structural basis for tau physiological function [74]. The central domain of tau is highly disordered and rich in proline [75]. The C-terminus of tau contains three (3R) or four (4R) MTBD due to the splicing of *MAPT* on exon 10. The number of MTBDs affects the microtubule-binding affinity and the propensity of tau to aggregate [76]. Under physiological conditions, the protein structure of tau possesses a large range of variation. A previous study showed that the soluble native tau has multiple conformations in cytoplasm [77]. The initiation of tau aggregation lies in its own structure, i.e., the two hexapeptide sequences in the second and third MTBDs, laying the foundation for fibrilization [78].

Pathological assembly of soluble tau and post-translational modification (PTMs) of tau may result in the formation of insoluble NFTs, leading to neuronal dysfunction such as synaptic dysfunction and altered mitochondrial trafficking [43, 79]. The starting point of tau aggregation is the dimerization through interactions between the two hexapeptide motifs VQIVYK (located at the beginning of the third MTBD, thus present in both 3R and 4R isoforms) and VQIINK (located at the beginning of the second MTBD, thus present only in 4R isoforms) [78, 80]. Dimerization of tau serves as the core for sequential nucleation and elongation [81]. Elongated oligomers further serve as the template for amyloid formation with β-sheet structure, which eventually forms paired helical filaments (PHFs), giving rise to highly structured polymorphs seen in NFTs [82]. Genetic mutations such as P301L [83], and PTMs such as multi-site hyperphosphorylation [84, 85], are believed to facilitate tau aggregation. For example, the P301L tau mutation results in alterations of the hexapeptides, making tau more susceptible to aggregation [83]. A diagram of mutations in *MAPT* and tau isoforms is illustrated in Fig. 1.

### Structural basis for α-syn aggregation

**Fig. 2:**
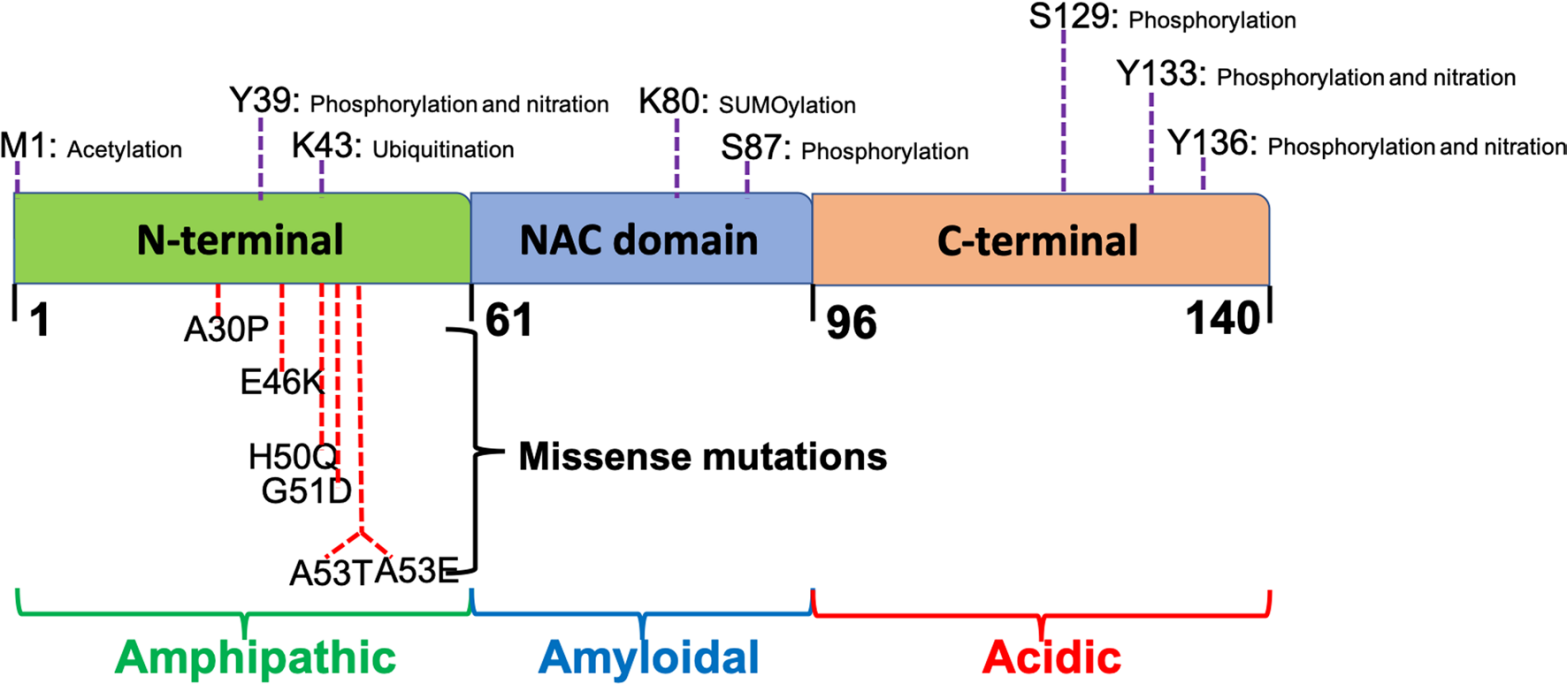
Structure, mutations and PTMs of α-syn protein. α-Syn has three domains: the amphipathic N-terminus, the non-amyloid-β component (NAC) which is prone to aggregate, and the acidic C-terminus. Common point mutations of α-syn are A30P, E46K, H50Q, G51D, A53T and A53E. The PTMs of α-syn mainly include phosphorylation, nitration, acetylation, ubiquitination and SUMOylation [43, 162]

**Fig. 3:**
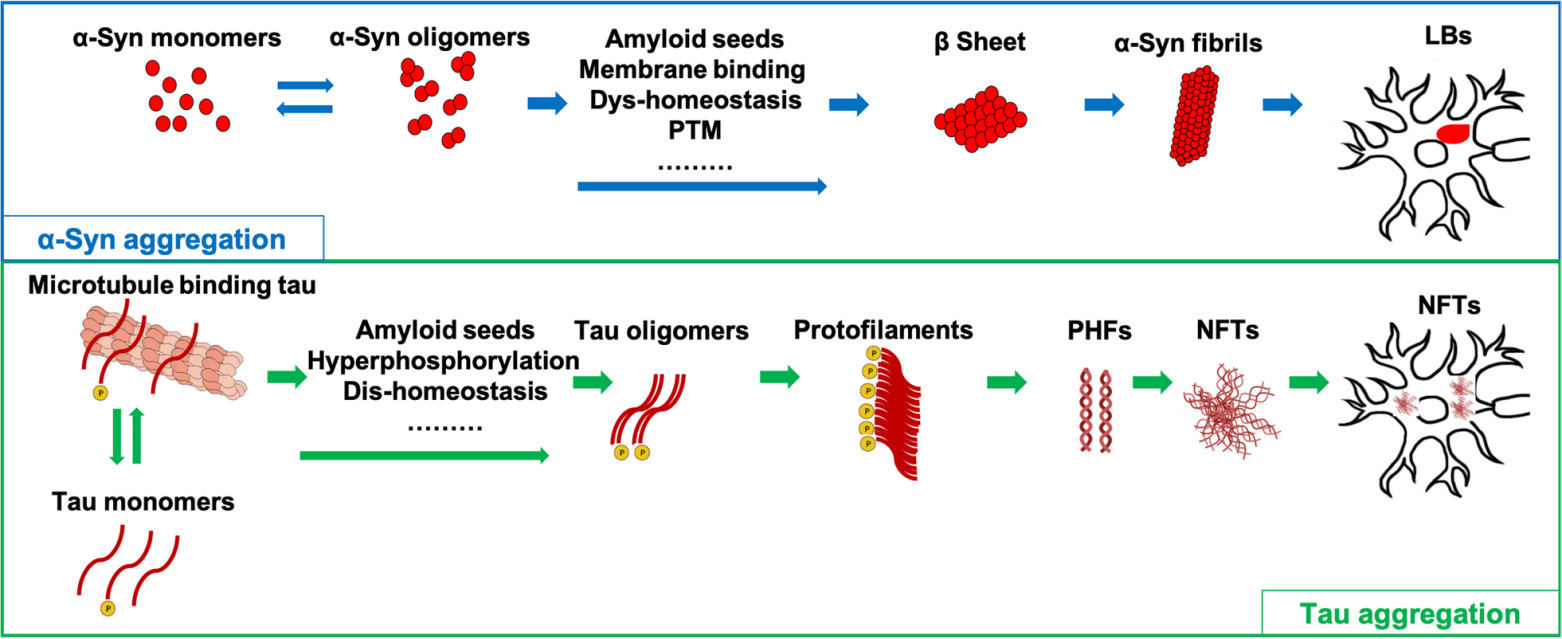
Processes of tau and α-syn aggregation. Under pathological conditions, α-syn accumulates and forms the β-sheet structure in the cytoplasm, which then becomes an amyloid fibrillary structure, the main component of LBs. On the other hand, tau monomers become hyperphosphorylated and form numeric molecules, which then aggregate into protofilaments and form PHFs, eventually becoming NFTs

α-Syn contains 140 amino acids encoded by the *SNCA* gene [86]. Little is known about the physiological functions of the protein. α-Syn has been shown to play a role in regulating synaptic neurotransmitter release [87]. The protein contains a N-terminal amphipathic region, a central amyloidal region and a C-terminal acidic domain (Fig. 2). The N-terminus of α-syn has lipid-binding properties and facilitates the initiation of aggregation. The central region is also named as the non-amyloid-β component core, which contains an amyloidogenic domain (61-95) [88]. The C-terminus is the structural basis for the unfolded nature of the protein [89]. α-Syn aggregation is partly due to its own natively unfolded structure and various pathogenetic *SNCA* mutations, as mentioned above [90]. Physiologically, the protein executes its neurological function in the cytoplasm in the forms of monomers and oligomers. Genetic instability, introduction of external amyloid seeds, dys-homeostasis of membrane structure, etc., can disrupt the equilibrium of α-syn monomers and oligomers, induce abnormal membrane binding and cause initiation of aggregation [91]. During the process of α-syn aggregate formation, PTMs play important roles (Fig. 2). Phosphorylation of α-syn at serine 129 is considered as a pathological marker of LB formation. About 90% of the α-syn extracted from synucleinopathy patient brains is phosphorylated at serine 129 [92]. Under pathological conditions, α-syn converts from the monomeric or oligomeric forms to the β-sheet structures. The β-sheets serve as the template and seed soluble monomers to generate protofibrils and eventually amyloid fibrils [91, 93]. The processes of tau and α-syn aggregation are illustrated in Fig. 3.

## Tau and α-syn pathology spreading

Postmortem studies of AD and PD have revealed the possibility of intercellular spreading of tau and α-syn, either to adjacent cells or between brain regions and peripheral organs [94–96]. Pathological staging using AD patient brain tissues has outlined a path of NFT deposition, beginning in the locus coeruleus and spreading to the entorhinal region and hippocampus, and eventually to the neocortex. The predictable manner of tau spreading correlates with the progress of cognitive decline [97, 98]. α-Syn spreads from the periphery to the brain following the Braak staging [99]. Typically, misfolded and aggregated α-syn in the dorsal motor nucleus of vagus can spread to other brain regions. Enteric deposition of LBs in early PD and incidental LB disease (iLBD) cases has also been reported [100], suggesting the propagating pathway from the peripheral enteric nervous system to the central nervous system (CNS) via the vagal nerve [101].

The cell-to-cell transfer of both tau and α-syn are hypothesized to follow a “prion-like” manner [102–107]. The molecular mechanisms by which the external aggregated seeds from donor cells enter recipient cells are still unknown. In the recipient cells, the seeds provoke the unfolding of the native monomeric proteins and initiate sequential aggregate formation. This is rather a process of dissemination than simple endocytosis and exocytosis.

The processes of tau and α-syn release and uptake are not fully understood. It is speculated that tau mainly spreads between cells through synaptic connectivity, rather than being predicted by proximity [98, 99]. Tau and α-syn both enter and exit cells through endocytosis, exosomes [91] and tunneling nanotubes [108, 109]. In addition, receptor proteins that mediate cell-to-cell transmission of both tau and α-syn have been reported. Lipoprotein receptor-related protein 1 (LRP1) has been shown to regulate the cellular entry of α-syn monomers and oligomers. Knock-out of LRP1 in mice significantly reduces the uptake of α-syn [110]. LRP1 is also a receptor for tau endocytosis, as blocking LRP1 significantly reduces tau uptake in neuroglioma cells and stem cell-derived neurons [111]. Lymphocyte activation gene 3 (LAG3) is necessary for receptor-mediated α-syn endocytosis [112]. Meanwhile, depletion of LAG3 can also decrease the uptake of tau by primary neurons [113].

When acting on cells, both tau and α-syn induce neuroinflammation and oxidative stress, which are common pathogenic features of neurodegeneration [114, 115]. For example, in primary microglial culture, preformed tau fibrils activate microglia in an NF-κB-dependent manner [116]. In addition, P301S tau transgenic mice show activation of astrocytes [117]. In synucleinopathies, longitudinal gene profiling studies have revealed the essential role of microgliosis in the early stages of PD, preceding the course of neurodegeneration [118]. Moreover, impairment in mitochondrial function [119, 120], synaptic transmission [121] and autophagy [122, 123] has been revealed in both proteinopathies.

The intercellular propagation of tau and α-syn pathologies follow preferential routes and share mechanisms such as exosomes and receptor-mediated endocytosis [124]. The similar spreading pathways and cellular toxicity between synucleinopathy and tauopathy may serve as a reference for developing pathology-targeting therapeutic interventions.

## Pathological continuum between tauopathies and synucleinopathies

### Co-occurrence of tau and α-syn pathology

Both tau and α-syn have a structural basis that confers an aggregation-prone property, leading to the typical pathological progress of tauopathy and synucleinopathy [86]. Co-presence of tau and α-syn has been shown in various diseases and models of neurodegeneration. Brain deposition of α-syn is found in more than 50% of AD patients [125, 126]. α-Syn was first identified in the AD brain in 1993 by Ueda et al*.*, which then was identified as a non-Aβ peptide fragment in the isolated Aβ plaques [127]. However, in later studies, the co-localization of α-syn was found to a greater extent with tau than with Aβ in AD brains [125]. Lippa et al*.* demonstrated that the insoluble forms of α-syn were present mainly in the amygdala of familial AD patients, some of which co-localized with tau NFTs [128]. In 145 sporadic AD patients examined in the study by Hamilton et al*.,* LBs were found in the brains of more than 60% patients, predominantly in the amygdala, rarely in the substantia nigra [126]. Uchikado et al*.* found that in 260 AD patients 62 cases had amygdala LBs, and in most of these cases, NFTs and LBs were found in the same cells [129]. The distribution pattern of LBs in AD patients differs significantly from that in PD, with absence of LBs in the brainstem, but more in the limbic and olfactory regions [130]. Tau fibrilization was also observed in PD patients carrying the A53T mutation [131]. Colocalization of tau and α-syn epitopes has been detected in LBs in multiple studies [132, 133]. In 80% cases of AD and diffused LB disease, tau immunoreactivity, especially phosphorylated tau, was spotted in LBs in the medulla [132]. In familial PD and DLB cases, PHF tau antibody can partially label LBs in the same neuronal cell, suggesting the co-occurrence of tau and α-syn in synucleinopathy brains [133]. The co-existence of tau and α-syn indicates the overlapping of pathology formation in both groups of disease.

### Tau/α-syn co-aggregates in pathology spreading

Compared to a singular administration of tau or α-syn, the existence of co-pathology of α-syn and tau has stronger effects in facilitating pathological spreading of the proteins. In mice injected with both AD patient brain lysates and α-syn fibrils, phosphorylated tau aggregation was significantly elevated in the presence of α-syn, and spread to broader brain regions such as the retrosplenial cortex and the supramammillary nucleus [134]. In vivo, transduction of tau and α-syn fibrils together induced a significant increase of insoluble tau pathology but not α-syn aggregates in neurons [134]. In addition, mice injected with tau-modified α-syn fibrils exhibited more severe α-syn pathology and faster spreading of α-syn in the striatum, compared with pure α-syn fibril injections. Knockout of tau attenuated the α-syn propagation and the accompanied mitochondrial toxicity [24]. In addition, PD patient-derived α-syn/tau oligomers administered in tau transgenic mouse brains accelerated tau oligomer formation and induced more severe neuronal loss, compared to administration of tau oligomers alone [135]. Hippocampal injection of the tau/α-syn hybrid fibrils exacerbated tau pathology transmission in a tauopathy mouse model, compared with pure tau or α-syn fibril administration. Interestingly, when tau/α-syn co-polymers were administered  to synucleinopathy mouse brains, no elevation of α-syn pathology propagation was observed compared to pure α-syn injection. It seems that the effect of tau/α-syn hybrid oligomers on pathology transmission is much stronger in tauopathy models than in synucleinopathy models, suggesting a more thorough cooperation of the two proteins in the tau seeding process, reflecting a more prominent effect of α-syn amyloidogenicity compared to tau [136].

### Possible mechanisms underlying the formation of tau and α-syn co-pathology

**Fig. 4:**
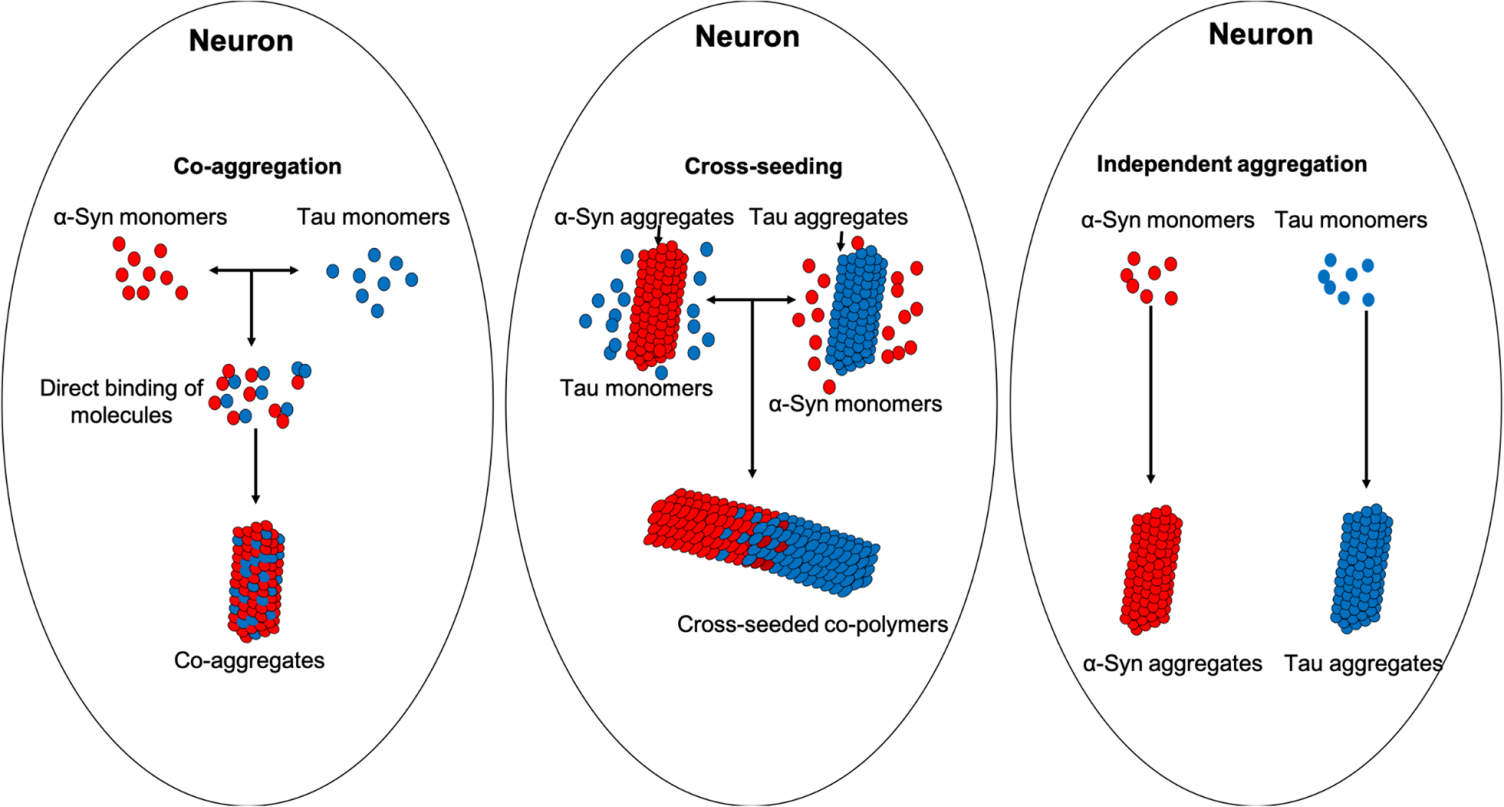
Mechanisms of co-pathology formation between tau and α-syn. We propose three possible models of tau and α-syn co-pathology formation in neurons. (1) Tau and α-syn monomers directly bind to each other and form co-aggregates (left), (2) tau or α-syn aggregates serve as the template and initiate elongation with monomers of the other protein, a process termed cross-seeding (middle); and (3) tau and α-syn aggregate independently from each other in the same neurons

Several studies have repeatedly proven the continuum of pathology between tau and α-syn. However, how the tau and α-syn co-pathology occurs is still unclear. Here we propose three hypothesized models for the molecular mechanisms underlying the tau and α-syn co-pathology based on current literature (Fig. 4).

First, direct binding between tau and α-syn monomers promotes the co-aggregation of proteins and initiates fibrilization. The proline-rich domain and the MTBD-containing region within the structure of tau protein directly interact with α-syn and presenilin-1, both regulating the formation of pathology in synucleinopathy [81, 137]. A recent study using a bimolecular fluorescence complementation system, which allows direct visualization of protein–protein interaction, revealed contacts between α-syn and tau at the molecular level, again laying the molecular basis for the formation of co-pathology [138]. Co-incubation of monomeric α-syn with tau variants promotes the amyloid formation of both tau and α-syn aggregates in vitro. NMR spectroscopy revealed that α-syn binds to tau molecules through its negatively charged C terminal [86]. Despite the direct binding, there is  also a study showing co-aggregation of tau and α-syn through liquid–liquid phase co-separation, by forming electrostatic complex coacervates, explaining the mechanisms of co-pathology formation from the biophysical perspective [139].

Second, tau and α-syn form co-polymers by means of templating and cross-seeding. The cross-seeding process follows a nucleation-dependent pathway, where one type of protein aggregate serves as a template, recruiting the monomeric, mutant or oligomeric form of the other [140]. From the biophysical perspective, oppositely charged proteins are more likely to cross-seed each other and the aggregation kinetics of co-aggregation is accelerated [141]. α-Syn and tau can cross-seed each other [142]. Adding modified polymorphic α-syn oligomeric strains to tau aggregates results in co-polymers with stronger propensity in pathology spreading [143]. Cryo-EM and solid-state NMR evidence from the study of Hojjatian et al*.* revealed that the α-syn aggregation in the presence of tau cofactor is related to specific polymorphs in the core region [144]. Meanwhile, in in vitro studies, α-syn fibrillary seeds induced intracellular inclusion formation of phosphorylated tau [145]. Studies in both neuronal culture and mice showed that distinct strains of α-syn preformed fibrils seed tau aggregation differently, suggesting the close association between templating and the sequential co-aggregate formation [24, 86, 146].

Third, in the post-mortem study by Ishizawa et al*.*, phosphorylated tau was present at the periphery of LBs instead of in an intertwining distribution pattern, in the brains of patients with Lewy body disease [132]. Nevertheless, Uchikado et al*.* showed that α-syn formed a central core surrounded by tau tangles in AD patient brains. Electron microscopy examination showed tightly packed tau filaments separated from the α-syn granule-patterned aggregates [129]. The finding of the co-pathology of tau and α-syn without cross-seeding of the two proteins suggests that α-syn and tau can aggregate independently in a same neuron.

### Perspectives

Despite the overlapping genetic risk factors and clinical manifestations, the co-pathology of protein aggregation, and the mutual facilitation of pathology spreading and cellular toxicity, there are divergent features between tauopathies and synucleinopathies. Intracellularly, tau is distributed predominantly in neuronal axons associated with microtubule, while α-syn is rather restricted to synaptic regions under physiological conditions [147, 148]. Moreover, transcriptomic studies have revealed that tau and α-syn variants have distinct cell type affinities. α-Syn is mainly present in neurons of the dopamine system, related to cellular functions such as synaptic transmission. Tau is predominantly located in the hippocampus and entorhinal cortex, related to cellular functions such as Ca^2+^ homeostasis [149, 150]. The propagation of tau aggregates is more limited to the CNS in contrast to the spreading pattern of α-syn from the periphery to the CNS [151]. However, studies by Beach et al*.* debated on the theory that α-syn pathology originates from peripheral organs. In their post-mortem analyses of synucleinopathy in iLBD, PD and normal elderly subjects, they found no presence of peripheral α-syn in subjects without brain α-syn pathology. This revoked the “body-first” hypothesis of α-syn aggregation [152, 153]. Unlike α-syn, it is hypothesized that the tau protein is present in the peripheral nervous system more as the “Big tau”, with a higher molecular weight due to the additional exon 4a, which makes the protein more strongly bind the cytoskeleton and less likely to undergo conformational changes and aggregation [154–156].

Based on the pathological continuum of tauopathies and synucleinopathies, common therapeutic interventions may be applied to both groups of diseases. Tau and α-syn share similar mechanisms of cell-to-cell transmission, including exosome release, receptor-mediated endocytosis, etc. Blocking the propagation process of both amyloid proteins could be a potential way to halt pathology progression and delay the development of diseases. Immunotherapies targeting extracellular tau and α-syn have been reported to be effective in clinical trials for disease modification, such as the passive-immunization antibody PRX002 [157] for α-syn and BIIB092 for tau [158, 159]. Preclinical studies have also reported some immunotherapeutic antibodies and active peptides that can effectively decrease protein aggregation and protect neurons [160, 161].

## Conclusion

We have summarized the clinical symptoms, genetic risks and pathological features of tauopathies and synucleinopathies. More importantly, we have discussed recent findings on the aggregation, propagation and toxicity of tau and α-syn, with a special focus on the pathological continuum of the two proteins and the two groups of proteinopathies. Tau and α-syn can cross-seed each other in the process of aggregate formation and spreading. The present review provides an update on disease classification of neurodegeneration and suggests niches of pathology intervention at early stages of tauopathies and synucleinopathies.

## Data Availability

Not applicable.

## Abbreviations

AD: Alzheimer’s disease
APOE: Apolipoprotein E
CBS: Corticobasal syndrome
CNS: Central nervous system
DLB: Lewy body dementia
FTD: Frontotemporal dementia
iLBD: Incidental Lewy body disease
LB: Lewy body
LN: Lewy neurite
MAPT: Microtubule-associated protein tau
MSA: Multiple system atrophy
NFT: Neurofibrillary tangle
PD: Parkinson’s disease
PDD: Parkinson’s disease dementia
PHF: Paired helical filament
PSP: Progressive supranuclear palsy
PTM: Post-translational modifications
α-Syn: α-Synuclein
